# Supplementary material for: A multiscale computational framework for the development of spines in molluscan shells
Source: PLoS Comput Biol. 2024 Mar 1;20(3):e1011835. doi: 10.1371/journal.pcbi.1011835 (PMC10936779; doi:10.1371/journal.pcbi.1011835)
Supplement: Attachment — Submitted filename: ResponseToReviewers.pdf [file pcbi.1011835.s002.pdf]

We thank the reviewers for their detailed reading of our manuscript and constructive feedback. We have revised the manuscript taking into consideration all the comments and recommendations. Our detailed responses to the reviewers' comments are given below. Associated changes in the revised paper appear in blue, with removed text appearing crossed-out in red. Other minor adjustments that helped with the natural incorporation of the required changes may not have been highlighted.

Reviewer 1:

The proposed framework is of interest to the scientific community as it represents a rare example of a multi-scale model for a biological process. Both models are simplistic to demonstrate the feasibility of the approach. However, the paper should clarify the biological plausibility of the obtained results, which is discussed in the conclusion. It would be beneficial to introduce this aspect more explicitly from the beginning. Additionally, the rationale for selecting mollusk shell growth as the specific use-case should be better explained.

We are pleased that the reviewer has appreciated our multi-scale approach. We have significantly restructured the introduction, in order to make more clear the purpose of the modeling, the motivation for studying spine formation in shells, and the limitations of the model.

For instance, the interconnection between the two models (lines 284-286) lacks justification and clarity. The choice of the chemical used and the impact of proportional concentration on the activator equation should be explored in detail, as it can significantly affect the observed behavior and serves as a crucial element in the multi-scale connection.

We agree, these choices most certainly impact the results. Given our simplified choice of chemical equation, with only an activator and substrate, it seems only natural to link the activator to the growth parameter. Of course, if we had used a different molecular equation, different links may have been possible. Exploring other possibilities would be straightforward; indeed this 'plug and play' aspect is one of the key features of our approach. But to actually input different models goes outside the scope (and point) of this work, in which we are more interested in presenting a framework of analysis than obtaining firm conclusions on the molecular interactions underlying mollusc spine formation.

In terms of how we explicitly define the link between the steady state activator profile a(x) and the growth parameters g1 and g2, our approach (described in Section 3 of the SM), was to find the best fit Gaussian profile to the activator, and then assign g2 based on the width, and g1 based on the amplitude. Since both systems are solved on the same spatial domain, the map for g2 is unambiguous. The only uncertainty arises in the scaling factor alpha that we use for g1=alpha a1, where a1 is the amplitude of the best fit Gaussian for a(x). For consistency, we used the same value of alpha for all simulations. This value was chosen based on some 'reverse engineering': the aggregated plots in Fig 4b already include the choice of alpha. A different alpha would simply serve to rescale the x-axis. But having done the fitting of g1-g2 to the shell images, we already knew that the relevant range of g1 is

between 0.1 and 1.0, hence the motivation for our choice was to obtain as many points as possible within the relevant range. In light of this scaling, it is evident that changing alpha by a small amount would only have a small quantitative effect on our results, shifting the best-fit {c1,c2,c3,c4} values; a larger change in alpha, though, would shift the data points completely out of the range of desired g1-g2. Taken together, these observations imply that the multiscale model is only capable of producing the right pattern if the link between scales is within a narrow range.

We thank the reviewer for noting this point, as we realize now that we did not sufficiently comment on this aspect. We have thus added a discussion on the choice of alpha to the SM, including how a change in alpha would impact our results.

Another aspect requiring clarification is the pre-integration of the tissue-level model for different pairwise combinations of parameters g1 and g2 (lines 341-342). While this approach reduces computational costs, it raises questions about the multi-scale nature of the study. It is unclear whether the molecular level can produce {g1, g2} values that were not generated, potentially resulting in different shapes than those used for evaluation from the zoo.

Indeed the molecular level model can produce a range of {g1,g2} values that were not matching those in the zoology of tissue-level shapes. We note that the axes in Figure 4, both (a) and (b), are continuous. Extending to pairs of {g1,g2} outside the range appearing in Fig 4 (a) leads to shapes that do not bear a resemblance to those observed in the real shells. Accordingly, those values will not be selected at the molecular scale, but are still useful to get a picture of the parameter space (Figure 4(b)).

To clarify this point, we have modified the text as follows:
"Rather than simulating the tissue model at each call within the optimization algorithm, we integrate before exploration the tissue-level model for different pairwise combinations of g1 and g2, thus producing a finite zoology of shape evolution curves that can be compared with real shells and providing a range of reasonable values for those parameters. This approach allows us to define both the quality and diversity for the exploration process: the quality of a given growth profile is defined as how close it matches a Gaussian shape and its diversity is defined as whether the corresponding {g1, g2} pair of that Gaussian is different enough from solutions found so far. Note that g1 and g2 are continuous values and thus will likely not match exactly the values used in the initial integration."

The use of the MAP-Elites algorithm for exploring parameter sets of the curve is not justified in the paper. It would be helpful to explain why this algorithm was chosen over other approaches such as parameter sampling (e.g., Latin Hypercube Sampling) and provide a rationale for its suitability in this context.

MAP-Elites is one of the most commonly used Quality-Diversity algorithms, a category of algorithms that has been well established to explore a high dimensional space and map it onto a phenotypic space. While the Latin Hypercube Sampling method could be used in the

current scenario (as we are only exploring over 4 parameters), it would not be able to scale to more complex cases. Based on the results from MAP-Elites (figure 4b), which provided a good coverage of the phenotypic space, we do not expect to find different results in the current case. We have however added that sampling method as an option in the code. A sample run of that method was significantly slower than the MAP-Elites approach, as it indiscriminately spent evaluations on areas of the parameter space that did not form Gaussian distributions. We have also added the following text to the main manuscript: "Quality-Diversity algorithms have been established as an efficient way to explore a high-dimensional space and build a fitness landscape across phenotypic characteristics. Note that for low dimensional problems other methods such as Latin Hypercube Sampling or Sobol sequence can efficiently deal with this exploration phase, which are also provided by our framework."

In section 2.3 (lines 289-290), the paper mentions fixing the diffusion coefficients due to the expectation of no variability between individuals. However, the impact of this choice is not addressed. Conducting a sensitivity study on these parameters would help justify such a simplification.

Thank you, this is a good point. We had fixed these values based on some informal simulations, sort of by trial and error. We have now conducted an analysis of the activator profile generated over a wide range of diffusion coefficients. This has produced a phase space diagram, categorizing the range of diffusion coefficients that give a reasonable pattern, and shedding some insight on how the pattern is impacted by activator and substrate diffusion. We also noted that while we have opted for a lower dimensional parameter space by fixing (d1,d2), our modeling analysis could in principle be conducted with varying diffusion.

Furthermore, in section 3.1, the impact of growth profile and spine shape is proposed, yielding interesting results. However, the methodology lacks details regarding how the values of the pair {g1, g2} were explored. Providing more information on this aspect is necessary to assess the quality of the obtained results.

The range of values correspond to those that produce a pattern comparable with the spines on real shells. Here we used some knowledge from previous works on spine formation, plus some sample simulations to find an appropriate range. We have added a few comments to this point in the SM.

Lastly, SM Section 5 lacks convincing fitting to biological data. While some of the curves match the observed photos, others deviate significantly (e.g., Figure 2: spines 2A, 4Aii, 5B, 10A; Figure 3: spines 6A, 7A, 15B; Figure 4: spines 8A and 9A). Since the spine fitting is done manually, it raises questions about the impact on the final results of the proposed methodology. It should be explicitly stated whether different experts would produce spines with similar values.

There are several specific features on the real spines that make the fitting process challenging: (1) the spines appear on the shell edge, which may be more or less curved; (2) many of the taller spines will have undergone self-contact (the two sides of the spine meet at

the center, beyond which there is a sort of ‘zippering up’ of the two sides; and (3) the tip of the spine, particularly for taller spines, folds back out of plane.
Each of these is a feature that does not exist in the mechanical model. While these might seem like small details that could be easily added, they are far from it. Spine formation on a curved edge would be perhaps the easiest feature to include, but the curvature is different for each spine, so there would be an entire extra dimension to the simulation. (The curvature is generally on the smaller side as well, so this feature is probably not especially important.) Self-contact is an especially tricky component to add to a mechanical model, as it includes complex issues such as transitions from a contact point to a contact region, additional unknowns (the location of the contact and jump in normal force), and brings severe issues with numerical convergence for integrating the mechanical boundary value problem, thus significantly slowing down computation time even if done correctly. Folding back may be even more complex of a detail to add, as it raises the system from two-dimensional to three. Some of the spines have also grown with a noticeably imperfect left-right symmetry (e.g. Jyogashima 15B); in such cases, we have tried to either balance the two sides or fit the growth curves to one side of the spine.

Despite these issues, we contend that the model generates a reasonable zoology for fitting to the real spines. And we did have these difficulties in mind in devising and executing the fitting process. In particular, we did not simply fit a single image from the model to the image of the shell. Rather, we imported the image of the shell to a GUI environment (Manipulate command in Mathematica), and stepped through the time sequence of evolving shell edges within the model, overlaid on the image. Here, we are aided by the fact that upon close inspection, each spine image has growth lines which may be interpreted as a time sequence of shapes of the developing spine. Thus, though we only show a single model output in the SM Figs, in fact we really compared a sequence of model shapes to a sequence of growth lines. In the cases of spines that displayed significant self-contact, the fitting process focussed on fitting to growth lines that occurred earlier in the development, before the self-contact. For spines that displayed significant folding back, we tried to take into account that the real shape has folded out of the plane of fitting. Since the spine image is taken at a point ‘beyond’ where the model can reasonably approximate the shape, it is inevitably the case that such spines do not, at a glance, look like very good fits.

While we are admittedly still picking the best fit by visual inspection, given the complexities outlined above, it is hard to imagine an automated process, e.g. using computer vision techniques, doing a better job. At the same time, we must acknowledge that the fitting of curves from the zoology to the spine images is not an exact science. Were someone else to perform the same exercise, we anticipate that there would be some differences in best fits, but not a dramatic change; i.e. it is unlikely that they would choose the exact same values for all shells, but the overall trend of a higher average g1 for the Jyogashima population would likely persist. This is why we have highlighted the general trend in the population difference: the generally higher curvature in the Jyogashima population seems visibly evident, and this is reflected in the fits, even if it is an imperfect science.

We thank the reviewer for highlighting this issue, as it has made us realize that we did not sufficiently explain these technicalities. We have thus significantly expanded our discussion of the fitting process in the SM.

Reviewer 2:

1. I am concerned about the usefulness of the current approach since the approach relies on many assumptions. There are many assumptions necessary before starting the optimization, and we need to select the following materials:
- reaction-diffusion equation: Gray-Scott model, activator-substrate model...
- cause of buckling: non-uniform growth, non-uniform mantle stiffness...
- what makes the spine shape different: genotype, phenotype, local environment...
- biochemical-mechanical coupling: an infinite number of combinations

The current conclusion can be drawn since we initially assumed that the dynamics are governed by the biochemical-mechanical coupling introduced in this paper. Since different sets of assumptions and equations would lead to different conclusions, I do not believe this approach can be used to understand the complex mechanism of shape formation. For example, there is a possibility that the spines of the two groups differ simply due to the local mechanical environment. I understood that the current framework can be used only when the assumptions are confirmed, which would be difficult to be achieved. I would like the authors to answer how they treat this problem in the manuscript.

We thank the reviewer for this comment. Indeed, there are many assumptions at play, and the conclusions are very much tied to the underlying assumptions at the different levels. However, we contend that this a ***feature*** *of our modeling, not a bug! In this manuscript, we are, as much as anything, presenting a modeling framework (this is why we have submitted the manuscript to the Methods Section of the journal). The framework has been designed to be "plug-and-play" and work with the assumptions of the team using it. We chose to demonstrate it with a specific multi-scale problem, and with specific underlying assumptions, as the reviewer points out.*

Ideally, the framework should be used as a way to compare multiple models and hypotheses. In principle, we could replace the activator-substrate model with another one, or replace the tissue mechanics model with an alternative description, and then analyze the difference in the parameter space generated (e.g., Fig 4 (b), Fig 5). The reason we have not done so is because (a) there is no obvious alternative candidate models that we should plug in, and (b) that is not really the point of the paper! The point is that if we were to change one of the sub models, the procedure for linking the models and analyzing their success at generating shapes that match the spine images would work in the same conceptual manner. It is that conceptual approach for linking the scales and comparing with real images that comprises the primary novelty in this work.

Having said that, we do want to be careful not to overstate the conclusions. In the end, yes, spine formation is a complex phenomenon. While the multiscale analysis we have presented, using the specific assumptions we have made, does generate what seems to be

a consistent story about the formation of spines in the different populations of T. sazae*, it would be taking it too far to conclude that this is exactly what is happening in these molluscs. We have thus gone through the paper and attempted to make the points above more clear, while also toning down any statements that could come across as firm conclusions about* T. sazae*. In particular, we have restructured the introduction in order to have a streamlined presentation of the purpose of the modeling, the 'plug and play' aspect, and reasonable expectations for the results.*

2. As far as I understood, the mechanical model introduced on page 9 was not used, and the morphoelastic rod is utilized to simulate the shape evolution. Why is the model on page 9 introduced?

We apologize for any inclarity in the text. The model introduced on page 9 is that of a morphoelastic rod. As such, it is indeed the model that is used in the rest of the text. We have clarified this by adding the

3. The manuscript was not friendly to readers not in this field since there were not enough explanations on the "secretion process" and "mantle" with schematics. There should be explanations of the technical terms with a simple schematic.

We have shifted some of the description of shell growth to the start of the methods section, where we have also added an expanded description of how the secretion process works. We considered adding a new schematic, but decided that the cartoon in Fig 2 b already captures the basic idea; we have pointed the reader to this schematic in the newly added description.

Minor Comments:
1. Line 128: no link to Reference -> Fixed
2. First paragraph of section 2.1.2: This paragraph is difficult to understand because the "biological facts" and "assumptions for the simulation" are explained at the same time. It would be helpful to clearly state the facts first and explain the assumptions afterward.

Thank you, we have reworded to try to make clear the biological process, as it is understood, and the modelling assumptions. We have also added a more detailed description of shell secretion in molluscs at the start of Section 2.

3. Line 249: z-axis was not defined.

Fixed.

4. Line 294: "to solving" to "to solve"

We believe the gerund 'solving' is correct in this instance.

5. Line 311: "there is a huge bottleneck at the tissue scale" does not make sense. The computational time of the tissue numerics is the bottleneck.

Thanks, we have reworded to clarify this.

6. Eq. (6): s and l are not defined.

We have added text to clarify that these are arc length parameters in the current configuration.

7. Line 340: "Rather than simulate" to "Rather than simulating"

Fixed

8. Line 341: define what is "pre-integrate"

This has been changed to “integrate before exploration”

9. Line 348: \hat{S} is not defined.

In fact, we are defining \hat{S} at this point in the text as a variable denoting the subset of parameters kept by the algorithm.

10. Line 462: I could not understand the sentence: "The ruggedness of the main cluster's bottom edge and the presence of a few points for low values of g1 and g2 tend to point towards the former possibility in that area". What is the former possibility in that area?

The former possibility refers to possibility (1) in the previous sentence. The text has been updated.

11. Line 463: numbers of g1 and g2 are not subscripts

Fixed

12. Line 480: previous Section

Thank you, this should not have been capitalised.

13. Line 497: Why is the sentence "Note that this does not..." parenthesized?

I think our intention was to view this sentence as a sort of aside, but on revisiting it makes more sense to not have parentheses, which we have now removed.

14. Line 499: What do you mean by "continues well past self-contact in a number of spines"?

We mean that in some cases, the spine continues forming after the two sides have come into contact. As the mechanical model is not suited for self-contact, all simulations are stopped once the two sides meet. In shells exhibiting self-contact, once the two sides come into contact, a sort of ‘zippering up’ occurs while the spine continues to grow taller. By construction, our model is not capable of capturing such morphologies well. We have tried to clarify this point in the text, and have also added a reference to SM Section 5, which includes an expanded discussion of self-contact.

15. Line 566: missing a period

Fixed

16. Figures in Appendix: Figure titles are only numbers.

We have changed these to "Supplementary Figure"

17. Equation number in Appendix: It should be like (A1) to avoid overlap with the main text

We have changed equation numbers to the (An) pattern.

18. Is the quality-diversity algorithm used only for molecular-level? or, also for tissue-level?

It is only for the molecular level.
